# De Novo *GLI2* Missense Variant in a Child With Isolated Hypopituitarism and Craniofacial Anomalies: Expanding the Phenotypic Spectrum

**DOI:** 10.1002/mgg3.70136

**Published:** 2025-09-04

**Authors:** Himanshu Goel, Katrina Harrison

**Affiliations:** ^1^ Genomic Health West Leederville Western Australia Australia; ^2^ Hunter Genetics Waratah New South Wales Australia; ^3^ University of Newcastle Callaghan New South Wales Australia

## Abstract

**Background:**

Culler–Jones syndrome (CJS) is an autosomal dominant disorder characterized by hypopituitarism, postaxial polydactyly, and craniofacial anomalies, associated with pathogenic *GLI2* variants. Genotype–phenotype correlations suggest missense variants may present with isolated pituitary phenotypes.

**Methods:**

We evaluated an 8‐year‐old boy referred for short stature, failure to thrive, and neurodevelopmental concerns. Clinical assessment, endocrine evaluation, imaging studies, and trio exome sequencing were performed.

**Results:**

The patient exhibited growth hormone deficiency, dolichocephaly, midline diastema, lip and tongue ties, hypotonia, and ADHD. No polydactyly was noted. Trio exome sequencing revealed a de novo heterozygous likely pathogenic *GLI2* variant (c.1496G>T; p.Arg499Leu) located within the DNA‐binding zinc finger domain.

**Conclusion:**

This case expands the phenotypic spectrum of *GLI2*‐related disorders and reinforces that non‐truncating *GLI2* variants are often associated with isolated hypopituitarism and subtle craniofacial or neurodevelopmental features. Genomic testing should be considered in similar clinical presentations.

## Graphical abstract

A novel de novo *GLI2* variant (p.Arg499Leu) was identified in a child with hypopituitarism and subtle craniofacial and neurodevelopmental features, broadening the clinical spectrum of *GLI2*‐related disorders and supporting genotype–phenotype correlations.

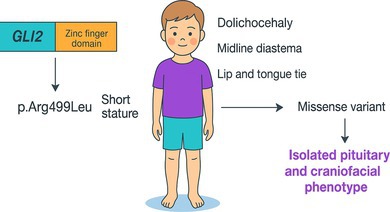

## 1 Background

Culler–Jones syndrome (CJS) (OMIM#615849) is a rare autosomal dominant disorder characterized by postaxial polydactyly, hypopituitarism, particularly growth hormone (GH) deficiency, and midline craniofacial anomalies (Culler and Jones 1984; Cohen 2012; Kevelam et al. 2012). It is part of the phenotypic spectrum associated with pathogenic/likely pathogenic (P/LP) variants in the *GLI2* (OMIM*165230), a zinc finger transcription factor involved in the Sonic Hedgehog (SHH) signaling pathway. The syndrome shows incomplete penetrance and variable expressivity, with overlapping features of holoprosencephaly (HPE) spectrum disorders (Franca et al. 2010; Bear et al. 2014; Niida et al. 2017; Martin‐Rivada et al. 2019; Valenza et al. 2019; Elward et al. 2020).

## 2 Case Presentation

We report an 8‐year‐old boy referred for evaluation of short stature, failure to thrive, hypotonia, and generalized hypermobility. He was first diagnosed with failure to thrive at 4 months of age. His birth weight was 3.34 kg, and length was 50 cm. There was a history of delayed closure of the anterior fontanelle. At 2 years of age, he was noted to have bilateral esotropia, for which he underwent corrective surgery. He also had a single episode of elevated alkaline phosphatase (1000 IU/L) and hyperphosphaturia of uncertain origin. Hyperphosphaturia resolved at the age of 8 years. Isolated phosphaturia was noted with persistently low renal phosphate threshold. He also had a history of recurrent otitis media requiring grommet insertion, mild bilateral hearing loss, and obstructive sleep apnoea (OSA).

There was no family history of similar features or known genetic conditions.

**FIGURE 1:**
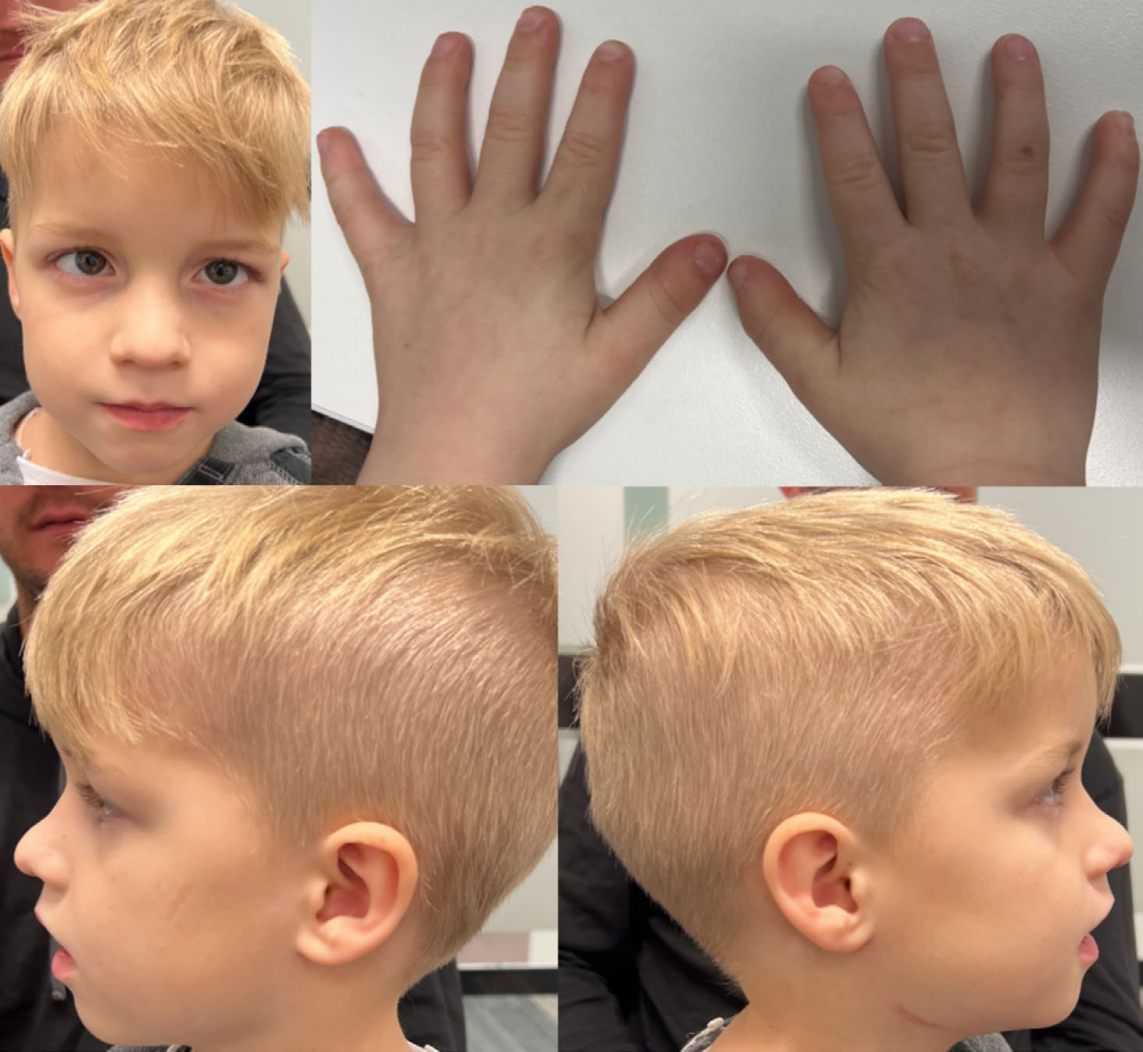
Seven years old boy has craniofacial findings that include dolichocephaly, prominent superficial veins, bilateral esotropia, microdontia, a midline diastema between the upper incisors, and the presence of both lip and tongue ties. Published with parental informed consent.

At 7 and a half years of age, his height was 104.5 cm (−3.7 SD), weight 16.9 kg (−2.7 SD), and head circumference 52 cm (45th percentile). He had dolichocephaly, prominent superficial veins, microdontia, a midline diastema between his upper incisors, and lip and tongue ties. (Figure 1, consent obtained). His growth velocity was 1.5 cm per year. He was started on recombinant growth hormone (rhGH) at 7 and a half years of age.

His gross motor milestones were achieved within the expected timeframe; however, physiotherapy and occupational therapy were required to address hypotonia and joint hypermobility. He exhibited speech articulation difficulties, academic delay, and a diagnosis of attention deficit/hyperactivity disorder (ADHD). Cognitive functioning was otherwise reported to be within normal limits.

Fragile X testing and SNP microarray were normal.

## 3 Methods

Trio exome sequencing was performed by Victorian Clinical Genetics Service, Melbourne, Australia, using Agilent SureSelect v7 exome capture and Illumina NovaSeq 6000 sequencing. Coverage exceeded 98% at 10×, and data were aligned to GRCh38 using a validated in‐house bioinformatics pipeline. Quality control included sex determination, contamination checks, and relatedness analysis. Variant annotation followed HGVS nomenclature and ACMG guidelines (Richards et al. 2015). A de novo heterozygous missense variant was identified in *GLI2* (NM_001374353.1:c.1496G>T; p.Arg499Leu). IGV screenshot validating the de novo status and zygosity are included in Figure S1A,B. This variant lies within the zinc finger DNA‐binding domain, affects a highly conserved arginine residue, and is absent from population databases (gnomAD v2, v3, v4), ClinVar, and HGMD. ACMG classification is PS2_moderate (confirmed de novo), PM1 (critical domain), PP3_strong (alphamissense = 0.999) and PM5_supportive (results in a different amino acid change than the known pathogenic variant) consistent with a “likely pathogenic” designation. No other clinically relevant variants were detected.

Endocrine testing included two GH stimulation tests (clonidine peak GH: 4.2 ng/mL; insulin peak GH: 3.8 ng/mL), low IGF‐1 and IGFBP‐3 levels, and normal results for cortisol, ACTH, TSH, FT4, LH, FSH, and prolactin. Bone age was delayed. Brain MRI showed normal size and structure of the anterior pituitary and no ectopic posterior lobe. A skeletal survey was essentially normal, although bone age was delayed. Endocrine testing showed normal cortisol levels. A pituitary MRI was normal.

One‐year follow‐up showed significantly improved growth velocity (10.5 cm/year) and a height SDS increase from −3.7 to −2.6.

## 4 Discussion

*GLI2* encodes a zinc finger transcription factor that plays a key role in the SHH signaling pathway, which is essential for embryonic development, particularly of the central nervous system midline structures, limbs, and pituitary gland (Roessler et al. 2003). Disease‐causing variants in *GLI2* have been associated with a spectrum of phenotypes, ranging from isolated pituitary hormone deficiencies to classic HPE and CJS, which is characterized by the triad of postaxial polydactyly, hypopituitarism, and craniofacial anomalies (Roessler et al. 2003). The phenotypic expression of GLI2‐related disorders is highly variable and often exhibits incomplete penetrance and intrafamilial variability (Elward et al. 2020). In mouse models, *Gli2* inactivation leads to pituitary agenesis and forebrain midline abnormalities, supporting its role in early embryonic development (Martin‐Rivada et al. 2019; Roessler et al. 2003; Arnhold et al. 2015).

In this report, we describe a child with features consistent with CJS who was found to have a *de novo* heterozygous missense variant in *GLI2*, c.1496G>T; p.(Arg499Leu). He had isolated GH deficiency, subtle midline craniofacial anomalies, neurodevelopmental challenges, but without polydactyly. This variant lies within the fifth zinc finger motif of the DNA‐binding zinc finger domain, a region critical for interaction with target DNA sequences. The arginine residue at position 499 is highly conserved across vertebrate orthologs. Substitution of this positively charged arginine with a hydrophobic leucine likely disrupts hydrogen bonding and DNA contact, akin to pathogenic variants at Arg499. Alphamissense and CADD scores (38.0) support a damaging effect. Several other missense variants affecting adjacent zinc finger residues (e.g., p.Arg462Gly, p.Arg499Pro, p.Glu501Lys, p.Tyr558His) have also been classified as likely pathogenic, further supporting the relevance of this domain (Babu et al. 2019; Corder et al. 2022).

Our patient did not exhibit polydactyly, reinforcing previous observations that missense variants, in contrast to truncating mutations, are more often associated with isolated pituitary phenotypes rather than the full CJS triad. In a review of 112 individuals from 65 kindreds with *GLI2* variants, only 37% of those with truncating variants had both pituitary anomalies and polydactyly, whereas non‐truncating variants were associated with a pituitary only phenotype in 92% of cases with incomplete penetrance (Bear et al. 2014; Zhang et al. 2023; Fan et al. 2023; Wang et al. 2023). A previously reported child with anosmia, partial GH deficiency, hypogonadotropic hypogonadism, and low bone mass carried a different missense variant in *GLI2* (p.Tyr176Cys), further illustrating this genotypic and phenotypic heterogeneity (Zhang et al. 2023; Kim et al. 2019).

Endocrine evaluation revealed peak GH levels of < 5 ng/mL on two independent stimulation tests, along with low IGF‐1 and IGFBP‐3 levels, meeting diagnostic criteria for GH deficiency. Baseline cortisol, ACTH, TSH, FT4, LH, FSH, and prolactin levels were within normal limits. Serial endocrine follow‐up is planned to monitor for the potential emergence of additional pituitary hormone deficiencies, as GLI2‐related hypopituitarism may evolve over time (Franca et al. 2010; Roessler et al. 2003). Notably, the patient's growth response to recombinant human GH (rhGH) was favorable, with a 1‐year posttreatment height velocity of 10.5 cm/year and an improvement in height SDS from −3.7 to −2.6. These findings contribute valuable longitudinal data to the limited body of literature on therapeutic outcomes in GLI2‐related GH deficiency.

Neurodevelopmentally, our patient exhibited hypotonia, joint hypermobility, ADHD, and mild speech and academic delays. Although intellectual disability is not a consistent feature of GLI2‐related conditions, neurodevelopmental challenges including speech and motor delays have been reported in several case series (Bear et al. 2014; Valenza et al. 2019). The combination of endocrine, craniofacial, and neurodevelopmental findings in this case underscores the pleiotropic nature of *GLI2* dysfunction and supports inclusion of CJS in the differential diagnosis of children presenting with syndromic short stature and midline anomalies.

This case also highlights the diagnostic utility of exome sequencing in cases where initial investigations including imaging and microarray are nondiagnostic. Identification of a novel, likely pathogenic variant in *GLI2* provided molecular confirmation of a clinically suspected disorder and informed endocrine surveillance and management. Given the expanding spectrum of GLI2‐related phenotypes, clinicians should maintain a high index of suspicion for GLI2‐associated conditions even in the absence of classic features such as polydactyly or HPE.

## 5 Conclusion

We report a novel de novo missense variant, p.Arg499Leu, in the zinc finger DNA‐binding domain of GLI2 in a child presenting with isolated GH deficiency, subtle midline craniofacial anomalies, and neurodevelopmental features without polydactyly. This case expands the phenotypic and mutational spectrum of GLI2‐related disorders and highlights the diagnostic value of exome sequencing in children with unexplained hypopituitarism and syndromic short stature. Our findings support ongoing endocrine surveillance for the potential evolution of additional pituitary deficits and contribute to the growing literature on genotype–phenotype correlations in non‐truncating *GLI2* variants. Future functional assays are warranted to elucidate the mechanistic impact of this variant on *GLI2* transcriptional activity.

## Author Contributions

**Himanshu Goel:** conceptualised and wrote the draft. **Katrina Harrison:** provided the clinical details.

## Ethics Statement

Ethical review and approval were not required for the publication of this case report in accordance with institutional and national guidelines.

## Consent

Written informed consent was obtained from the patient's legal guardian for publication of the clinical details and any accompanying images.

## Conflicts of Interest

The authors declare no conflicts of interest.

## Supporting information


**Figure S1:** (A) A heterozygous missense variant was identified in the proband in *GLI2* (NM_001374353.1:c.1496G>T; p.Arg499Leu). IGV screenshot shows that this was absent in his parents. (B) p.Arg499 residue was highly conserved in top 100 vertebrate species.

## Data Availability

The data that support the findings of this study are available on request from the corresponding author. The data are not publicly available due to privacy or ethical restrictions.
