# Supplementary figures and images for: De Novo GLI2 Missense Variant in a Child With Isolated Hypopituitarism and Craniofacial Anomalies: Expanding the Phenotypic Spectrum
Source: Mol Genet Genomic Med. 2025 Sep 4;13(9):e70136. doi: 10.1002/mgg3.70136 (PMC12411262; doi:10.1002/mgg3.70136)

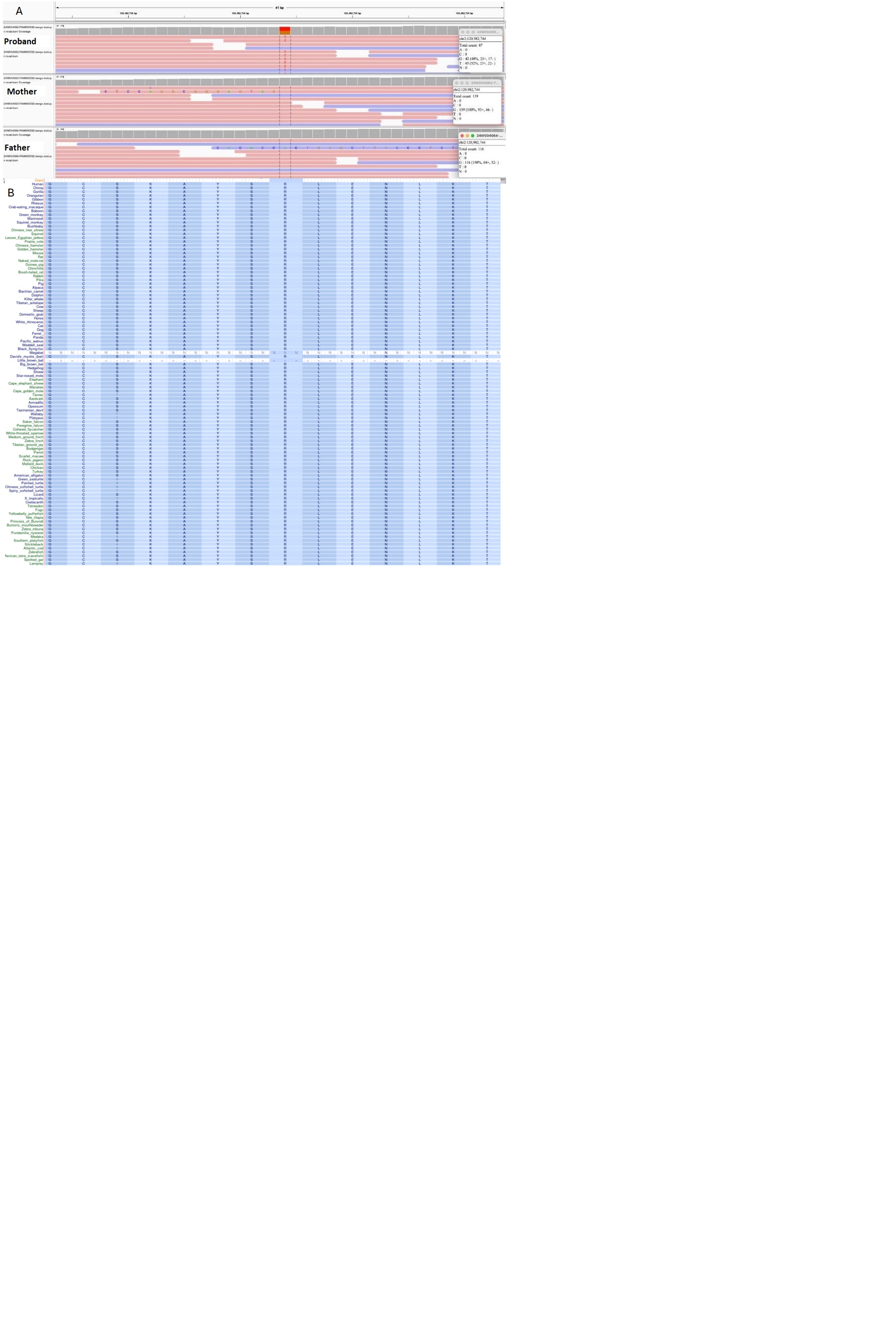

Supplement: Supplementary file 1 — Figure S1: (A) A heterozygous missense variant was identified in the proband in GLI2 (NM_001374353.1:c.1496G>T; p.Arg499Leu). IGV screenshot shows that this was absent in his parents. (B) p.Arg499 residue was highly conserved in top 100 vertebrate species. [file MGG3-13-e70136-s001.jpg]
